# Socioeconomic status and improvement in functional ability among older adults in Japan: a longitudinal study

**DOI:** 10.1186/s12889-019-6531-9

**Published:** 2019-02-19

**Authors:** Airi Amemiya, Naoki Kondo, Junko Saito, Masashige Saito, Daisuke Takagi, Maho Haseda, Yukako Tani, Katsunori Kondo

**Affiliations:** 1 Department of Health Education and Health Sociology, The University of Tokyo, 7-3-1 Hongo, Bunkyo-ku, Tokyo, 113-0033 Japan; 2 Department of Health and Social Behavior, The University of Tokyo, 7-3-1 Hongo, Bunkyo-ku, Tokyo, 113-0033 Japan; 3 Faculty of Social Welfare, Nihon Fukushi University, Aichi, Japan; 4 Department of Global Health Promotion, Tokyo Medical and Dental University, Tokyo, Japan; 5 Department of Social Preventive Medical Sciences, Center for Preventive Medical Sciences, Chiba University, Chiba, Japan; 6 Department of Gerontological Evaluation, Center for Gerontology and Social Science, National Center for Geriatrics and Gerontology, Aichi, Japan

**Keywords:** Disability, Socioeconomic status, long-term care

## Abstract

**Background:**

Recovery from functionally disabled status is an important target of public health measures for older adults. This study aimed to examine socioeconomic inequalities in the improvement of functional ability among older adults stratified by the level of disability at baseline.

**Methods:**

In the Japan Gerontological Evaluation Study, we conducted a mail survey of community-dwelling older adults (1937 men and 2212 women) who developed functional impairment during 2010–2014. The survey data were individually linked to the longitudinal records of changes in the levels of functional disability based on the Public Long-Term Care Insurance System.

**Results:**

The mean (standard deviation) follow-up period was 316 (269) days. During follow-up, 811 participants (19.5%) showed improved functional ability. Among those with severe disabilities at baseline, men with 13 or more years of education were more likely to improve functional ability than men with 9 or fewer years of education (hazard ratio: 1.97, 95% confidence interval: 1.12–3.45). A similar association was observed among women (hazard ratio: 2.16, 95% confidence interval: 1.03–4.53). Neither income nor occupation was statistically associated with improved functional ability.

**Conclusions:**

There are education-related inequalities in the improvement of functional ability, especially among older adults with severe disabilities. Health policy makers and practitioners should consider the educational background of individuals with reduced functionality in formulating strategies to improve their functional ability.

**Electronic supplementary material:**

The online version of this article (10.1186/s12889-019-6531-9) contains supplementary material, which is available to authorized users.

## Background

More than a third of older adults aged ≥60 years experienced functional disability worldwide in 2004 and the prevalence of disability has been increasing due to an aging population [1]. The improvement of functional ability is considered important because of its impact on the quality of life of older adults and the demand for long-term care (LTC) services [2]. In Japan, 6.0 million individuals were certified as eligible to use public LTC insurance (LTCI) benefits in 2015. The LTCI benefit expenses were 9.0 trillion Japanese yen (equivalent to approximately 80.0 billion US dollars) [3]. Although the majority of disabilities were progressive in the long-term, 1 in 10 individuals had improved functional ability within a year [4]. The trajectory of functional ability among older adults may improve if they receive adequate health care [2].

Factors affecting the trajectory of functional ability among older adults include physical, psychosocial, and socioeconomic factors [5–9]. In this study, we have focused on socioeconomic status (SES) as an important target of public health interventions aiming to achieve equity in health and longevity [2, 10]. However, the evidence for the association between SES and the improvement of functional ability is inconsistent [8, 11–14]. A longitudinal study in the UK [11] reported that those with higher education had higher rates of recovery from morbidity/disability independent of comorbidity, with a follow-up interval of 2–10 years. In contrast, other studies from the US [8], Europe [12, 13], and Taiwan [14] reported neither education- nor income-related inequalities in the improvement of functional ability after the onset of disability. These mixed results could partly be due to the difference in net and gross effects of SES on functional ability, adjustment for other dimensions of SES in the analysis, and self-reported measurements of disability [15]. Potential mechanisms to explain how socioeconomic inequalities contribute to improvements in functional ability may include material and psychosocial pathways [16, 17]. Accessibility to health care services and rehabilitation programs may be limited among those with a low SES [16]. Moreover, studies have suggested that persistent psychological stress and lack of social support are likely to be greater among individuals with low SES, which are also barriers to improving physical ability [16].

Therefore, this study aimed to examine the independent contributions of three major proxy measures of SES, namely, educational attainment, income, and occupation to improving functional ability using a large Japanese population-based dataset linked to Japan’s LTCI database. The LTCI database includes objective measurements of the level of disability with dates of application for LTC service utilization and dates of changes in the level of disability.

## Methods

### Study population

The analyses were based on the Japan Gerontological Evaluation Study (JAGES) linked to the LTCI database of Japan. A description of the JAGES study design is reported elsewhere) [22]. In brief, self-administered questionnaires were mailed to community-dwelling older adults (aged ≥65 years) without physical or cognitive disabilities in 31 municipalities in 2010 (Additional file 1). The LTCI database was obtained from 24 municipalities in which a survey was conducted in 2010 as part of the JAGES program. The LTCI database includes information on the level of disability, the dates on which the level of disability changed, and the dates of death or movement to a different municipality. The period covered by the LTCI database ranged from 14 to 46 months depending on the municipality. Among the JAGES participants living in 24 different municipalities, 4239 individuals were newly certified to receive LTCI benefits during the survey period. The follow-up period began at the initial certification date for each participant. The follow-up period ranged from 1 to 1318 days. Those with missing data on age and gender (*n* = 85) and those whose follow-up periods were 0 (*n* = 5) were excluded, resulting in an analytic sample of 4149 subjects.

### Measurements of the level of disability

The level of disability was evaluated at the time of LTC service application and certification according to nationally standardized criteria (Additional file 2: Table S1) [18–20]. The Certification Committee for Long-Term Care Need in each municipality, consisting of physicians and other health experts, assigned the level of disability based on both home visit interviews and the opinions of the primary physicians. The level of disability was repeatedly measured within 6 months for the second assessment and at least once a year for following assessments according to government recommendations or measured at the time of reassessment for LTC service users or their families.

The level of disability was divided into seven categories: requiring support-1 and -2 and requiring LTC-l to LTC-5. The measurements were used in recent studies, which investigated the determinants of functional disability and their association with mortality [21–24]. The study population included only those who were assigned to the requiring LTC-l to LTC-5 groups at the time of the initial assessment, because those who were assigned to the requiring support-1 and -2 groups were only eligible for LTC prevention programs and not actual physical care. We categorized the study population into three disability groups according to the initial level of disability: mild (requiring LTC-1), moderate (requiring LTC-2 or LTC-3), and severe (requiring LTC-4 or LTC-5). Improvement in the level of disability was defined as an improvement of ≥1 level(s) during the follow-up period compared with the level at the time of the initial assessment. Improvements in the level of disability included transitioning into requiring support-1 and -2.

### Socioeconomic status

SES and other covariates were assessed in 2010 using the JAGES self-reporting mail-in questionnaires. Educational attainment was measured as completed years of schooling (≤9, 10–12, and ≥ 13 years). Annual household income was reported in 15 predetermined categories (in thousands of Japanese yen). The equivalized income was calculated by dividing each response by the square root of the household size. Household income was then categorized into quartiles. The occupation engaged in for the longest period of time was reported in 7 categories and, following recent studies, we dichotomized the occupations into manual (craft and related trade workers and skilled agricultural, forestry, and fishery workers) and non-manual (professionals, managers, clerical support workers, service and sales workers, and other non-manual workers) categories [25].

### Potential confounders

Comorbidities included the following self-reported chronic diseases: heart disease, stroke, diabetes, and hypertension [26, 27]. Depressive symptoms were measured using the validated Japanese short version of the Geriatric Depression Scale with a cut-off point of 5 (depressive [score: 5–15] and non-depressive [score: 0–4]) [27–29]. Other potential confounders measured at baseline included age at initial LTC certification, marital status, living status, and municipality [30–33]. Missing values were treated as dummy variables.

### Statistical analyses

We used the Cox proportional hazards model to assess the association between SES indicators (educational attainment, income, and occupation) and improvements in functional ability during the follow-up period. We evaluated the proportional hazards assumption by visual and statistical means. Time to improvement in the level of disability is measured in dates. Participants who died or moved to another municipality were censored from the date of their death or move to another municipality. In addition to the crude model, a second model was used with adjustments for age, gender, other SES, marital status, living status, comorbidities, depressive symptoms, and municipality. The analysis was stratified by gender and disability group, because SES has differential effects on functional transitions depending on the individuals’ prior functional state [34]. As for sensitivity analysis, we restricted our sample to those who were followed-up for > 6 months, because the second assessment of the level of disability was mostly conducted 6 months after the initial assessment. We applied the missing indicator method (including a dummy variable for missing data in the analysis) in our study, following recent recommendations [35, 36]. All statistical analyses were conducted using STATA (version 14.0; StataCorp, College Station, TX, USA). A *P* < .05 was considered statistically significant.

## Results

The mean ± standard deviation follow-up period was 316 ± 269 days. The maximum follow-up period was 1318 days. The mean ± standard deviation age at initial LTC certification was 81.5 ± 6.7 years. Among the study population, approximately half of the participants were women, more than half reported completed years of education of ≤9, and approximately a third reported their occupation as manual (Table 1). The proportion of individuals with improved functional ability during the follow-up period in the mild, moderate, and severe disability groups were 11.7, 24.4, and 25.4%, respectively. The characteristics of the cohort stratified by gender and disability group at the time of the initial assessment are shown in Additional file 3: Table S2.

**Table 1:** Characteristics of the Participants

Characteristic	All	Improved Functional Ability
	*n* = 4149	*n* = 811 (19.5%)
Gender, n (%)		
Men	1937	346 (17.9)
Women	2212	465 (21.0)
Education (years), n (%)		
≤9	2227	441 (19.8)
10–12	945	172 (18.2)
13+	460	100 (21.7)
Unknown	517	98 (19.0)
Income (quartiles), n (%)		
Q1 (lowest)	739	142 (19.2)
Q2	740	139 (18.8)
Q3	640	109 (17.0)
Q4 (highest)	701	151 (21.5)
Unknown	1329	270 (20.3)
Occupation, n (%)		
Manual	1551	316 (20.4)
Non-manual	1145	219 (19.1)
Unknown	1453	276 (19.0)
Marital status, n (%)^a^		
Single/divorced/widowed	1454	319 (21.9)
Married/cohabiting	2330	424 (18.2)
Unknown	365	68 (18.6)
Living alone, n (%)		
N	3281	635 (19.4)
Y	490	103 (21.0)
Unknown	378	73 (19.3)
Comorbidity, n (%)^b^		
0	1029	219 (21.3)
≥1	2408	474 (19.7)
Unknown	712	118 (16.6)
Depressive symptoms, n (%)		
N	1605	314 (19.6)
Y	1427	263 (18.4)
Unknown	1117	234 (20.9)
Disability group, n (%)^c^		
Mild (requiring LTC-1)	1653	194 (11.7)
Moderate (requiring LTC-2/3)	1605	391 (24.4)
Severe (requiring LTC-4/5)	891	226 (25.4)

^a^Single = never married

^b^Comorbidity = heart disease, stroke, diabetes, or hypertension

^c^Disability group = level of disability at the time of the initial assessment

*LTC* long-term care, *N* no, *Q* quartile, *Y* yes

Among men with severe disabilities, the hazard ratio for improved functional ability in those with ≥13 years of education versus those with ≤9 years of education was 1.91 (95.0% confidence interval [CI]: 1.17–3.12) in the crude model and 1.97 (95.0% CI: 1.12–3.45) after adjusting for other covariates (Table 2). There was no significant association between education and improved functional ability among men with mild and moderate disabilities. There was neither significant association between income nor occupation and improved functional ability among all 3 disability groups among men.

**Table 2:** Hazard Ratios for Improved Functional Ability Among Men According to Socioeconomic Status

Socioeconomic Status	Disability Group at the Time of the Initial Assessment					
	Mild (*n* = 664)		Moderate (*n* = 789)		Severe (*n* = 484)	
	Crude	Model^a^	Crude	Model^a^	Crude	Model^a^
	HR (95.0% CI)	HR (95.0% CI)	HR (95.0% CI)	HR (95.0% CI)	HR (95.0% CI)	HR (95.0% CI)
Education (years)						
≤9	Ref.	Ref.	Ref.	Ref.	Ref.	Ref.
10–12	0.81 (0.43–1.53)	0.87 (0.45–1.69)	1.00 (0.69–1.45)	1.15 (0.77–1.71)	0.84 (0.49–1.44)	0.86 (0.49–1.51)
13+	1.51 (0.80–2.85)	1.58 (0.79–3.15)	0.88 (0.57–1.37)	1.10 (0.68–1.78)	1.91 (1.17–3.12)^**^	1.97 (1.12–3.45)^*^
Income (quartiles)						
Q1 (lowest)	Ref.	Ref.	Ref.	Ref.	Ref.	Ref.
Q2	0.93 (0.43–1.98)	0.89 (0.39–2.01)	0.84 (0.50–1.39)	0.80 (0.46–1.37)	1.22 (0.64–2.32)	1.10 (0.54–2.26)
Q3	0.95 (0.41–2.20)	1.00 (0.40–2.46)	0.75 (0.44–1.28)	0.71 (0.40–1.27)	0.94 (0.46–1.93)	0.93 (0.42–2.09)
Q4 (highest)	1.40 (0.66–2.95)	1.64 (0.74–3.65)	0.84 (0.49–1.42)	0.76 (0.44–1.31)	1.20 (0.62–2.32)	1.02 (0.47–2.22)
Occupation						
Manual	Ref.	Ref.	Ref.	Ref.	Ref.	Ref.
Non-Manual	0.95 (0.57–1.59)	1.02 (0.57–1.82)	0.80 (0.56–1.13)	0.78 (0.53–1.15)	1.27 (0.81–2.01)	1.19 (0.71–2.00)

^**^P < .01, ^*^P < .05. ^a^Adjusted for age, other socioeconomic status, marital status, living status, comorbidities, depressive symptoms, and municipality. CI, confidence interval; HR, hazard ratio; Q, quartile; Ref., referent category. Estimates for missing categories of SES indicators were not reported

Among women with severe disabilities, the hazard ratio for improved functional ability in those with ≥13 years of education versus those with ≤9 years of education was 1.39 (95.0% CI: 0.73–2.62) in the crude model and 2.16 (95.0% CI: 1.03–4.53) after adjusting for other covariates (Table 3). Although education was associated with the improvement of functional ability, there was no specific association between income and improved functional ability among women with severe disabilities (*P* for trend = .570). Occupation-related differences in improved functional ability were not significant across all 3 disability groups among women.

**Table 3:** Hazard ratios for Improved Functional Ability Among Women According to Socioeconomic Status

Socioeconomic Status	Disability Group at the Time of the Initial Assessment					
	Mild (*n* = 989)		Moderate (*n* = 816)		Severe (*n* = 407)	
	Crude	Model^a^	Crude	Model^a^	Crude	Model^a^
	HR (95.0% CI)	HR (95.0% CI)	HR (95.0% CI)	HR (95.0% CI)	HR (95.0% CI)	HR (95.0% CI)
Education (years)						
≤9	Ref.	Ref.	Ref.	Ref.	Ref.	Ref.
10–12	0.98 (0.64–1.52)	1.03 (0.65–1.63)	0.95 (0.68–1.34)	0.98 (0.68–1.41)	1.16 (0.74–1.83)	1.32 (0.81–2.18)
13+	0.67 (0.31–1.45)	0.63 (0.28–1.41)	1.10 (0.67–1.81)	1.24 (0.74–2.10)	1.39 (0.73–2.62)	2.16 (1.03–4.53)^*^
Income (quartiles)						
Q1 (lowest)	Ref.	Ref.	Ref.	Ref.	Ref.	Ref.
Q2	1.14 (0.62–2.08)	1.20 (0.65–2.24)	0.72 (0.45–1.14)	0.78 (0.48–1.29)	1.89 (0.97–3.66)^+^	2.12 (1.04–4.34)^*^
Q3	0.87 (0.44–1.72)	0.89 (0.43–1.82)	0.96 (0.60–1.51)	1.09 (0.67–1.79)	1.08 (0.53–2.19)	0.94 (0.45–1.98)
Q4 (highest)	1.21 (0.66–2.23)	1.21 (0.65–2.27)	0.83 (0.53–1.29)	0.92 (0.57–1.49)	1.85 (1.06–3.22)^*^	1.66 (0.92–3.00)^+^
Occupation						
Manual	Ref.	Ref.	Ref.	Ref.	Ref.	Ref.
Non-Manual	0.94 (0.57–1.55)	0.97 (0.57–1.67)	0.88 (0.62–1.27)	0.81 (0.54–1.20)	0.74 (0.46–1.18)	0.65 (0.38–1.12)

^**^P < .01, ^*^P < .05, ^+^*P* < .10. ^a^Adjusted for age, other socioeconomic status, marital status, living status, comorbidities, depressive symptoms, and municipality. CI, confidence interval; HR, hazard ratio; Q, quartile; Ref., referent category. Estimates for missing categories of SES indicators were not reported

When the analytic sample was restricted to those with a follow-up period of ≥6 months, similar associations were observed though the 95.0% CIs were wide due to the limited sample size (Additional file 4: Table S3 and Additional file 5: Table S4).

## Discussion

The results of our study show that education is positively associated with improved functional ability, especially among older adults with severe disabilities at the time of the initial assessment. Our findings were inconsistent with recent studies in other countries that have mostly shown no significant educational differences in the improvement of functional ability [8, 12, 14]. This inconsistency may be due to differences in the measurements of functional ability and follow-up intervals between the current study and other recently published reports [12, 14]. The current study used objective measurements, which assessed the initial level of disability at the time of the application for LTC service utilization and tracked the dates when the level of disability changed. Therefore, the current study could capture temporal changes in functional disability in each of the 3 disability groups. Other studies often defined disabilities as severe restrictions in activities of daily living, with follow-up intervals of 1–10 years, and did not stratify the analysis by disability severity.

Several mechanisms may explain how education contributes to improvements in functional ability. First, educational attainment is a predictor of socioeconomic status in later life, including occupation and income [16, 37–39]. Therefore, educational attainment may reflect the material conditions contributing to recovery: access to medical and rehabilitation services. Although in Japan most medical and rehabilitation services are covered by public health and LTC insurances, the existence of co-payment and other ancillary costs, including transportation and other opportunity costs may discourage them from utilizing those services regularly. In this study, however, inequalities in the improvement of functional ability were not clear for the levels of income and occupational classes. This may be related to the measurement issues in income and occupation in older ages. Previous studies have suggested that income was less predictive of health than wealth in older ages [40–42]. Moreover, our measure, the occupation individuals had engaged in for the longest period of time, may not be strongly linked to the current living arrangements of older adults, because the majority of subjects had already retired. Occupation may not represent material conditions among older women adults in Japan, given that the majority of older households were man breadwinner households [43].

Poor education may also alter health behaviour due to the limited health literacy and psychosocial stress, which may be other pathways linking educational attainment and recovery. For example, financial strain, lack of engagement in social networks, and lack of social support could impede recovery [16, 37, 44]. Psychosocial stress may also lead to adverse health behaviours, such as smoking and alcohol abuse, which are known to be more common among less educated people [16]. The analysis of Japan’s nationally representative data revealed that low education was associated with limited health literacy that were required to evaluate and utilize health information in critical and communicative ways [45].

Education-related inequalities in the improvement of functional ability were not observed in the mild and moderate disability groups. One possible explanation was low statistical power, because fewer individuals improved their functional ability in the mild disability group than in the severe disability group. Alternatively, differences in disease structure by the levels of disability may explain the gap. In Japan, the most common reasons for requiring care among the mild and moderate disability groups were dementia (26%), stroke (16%), and frailty (13%) [3, 46]. The trajectories of dementia and frailty status often slowly or steadily declined without remission [47]. Therefore, it may be difficult, even for individuals with high SES, to improve their functional ability [47]. In the severe disability group, the most common reasons for requiring care in Japan included stroke (27%), dementia (23%), and bone fractures (11%) [3, 46]. The trajectory of functional ability of organ failure, including stroke or bone fractures, sometimes achieve a remission [47]; therefore, individuals with severe disabilities who have high SES may be able to improve their functional ability better than those with severe disabilities who have low SES, because of access to medical and LTC services [38, 48].

This study has several limitations. First, improvements in functional ability may be underestimated in this study. Japan’s public LTC system requires users to take the clinical examination of disability levels every 6 months and to consider the renewal of the disability level unless requested by the users before 6 months have passed. This could apply negative incentives for the prior half year request for the LTC service users who believe their functional ability has improved because, if they are classified as less disabled, the maximum amount of LTC cost coverage is reduced. This results in a delay in capturing improvements in disability. Second, the LTCI database used in this study does not include information concerning the disqualification from LTCI eligibility due to regain of functional independence. However, the impact of this missing information may be small because the proportion of individuals who were disqualified over 4 years was < 3%. This may lead to an underestimation of the improvements in functional ability [49]. Third, the LTCI database may not capture changes in the level of disability after hospital admission, because LTC services are not used during hospitalization. Fourth, income may not capture the socioeconomic status of older adults, given that they are likely to rely on pension, savings, and other assets they have. Therefore, other measures such as degree of wealth should also be investigated. However, the questionnaires used in the current study did not include information on wealth. Degree of wealth may be more strongly associated with the health of older adults than income, because wealth indicates the ability to meet sudden expenditures such as medical expenses; however, measurement of wealth might be more difficult than that of income because wealth includes multiple factors [41, 50]. Therefore, the results of the current study, using income as a measurement of SES, might be underestimated. Fifth, the LTCI database used in this study did not include information on the major causes of functional disability. The aetiology in the course of functional disability might be different based on the cause of the initial onset of functional decline, and the estimates based on these causes may have more valuable clinical implications. The course of functional disability based on its causes warrants further study [47]. Sixth, some potential confounders that we considered, such as comorbidity, could be mediators linking SES to our health outcome. However, in our preliminary analysis, we evaluated the changes in the point estimates of the SES/outcome associations in the models including or excluding those factors step-by-step, and confirmed that changes in point estimates are not large. Finally, we applied the missing indicator method to address missing data on our explanatory variables; another approach such as multiple imputation could be an alternative, though the application of such methods to complex longitudinal merged data has been under debate [51, 52].

## Conclusions

Despite these limitations, our study suggests that education-related inequalities among older adults have important policy implications. In Japan, there are systems of free or low cost medical and LTC services for older adults with financial difficulties [53, 54]. However, these systems do not consider other social disadvantages, including low educational attainment. To reduce health inequalities among older adults with disabilities it is important to consider various socioeconomic backgrounds of older adults, and to support them according to the extent of their need [55].

## Additional files

Additional file 1: Survey Questionnaire (Japan Gerontological Evaluation Study, version 2010) (DOCX 43 kb)

Additional file 2: **Table S1.** Japan’s Long-Term Care System: Criteria and Benefit Limits (Standard Amounts) for In-Home Services (DOCX 15 kb)

Additional file 3: **Table S2.** Characteristics of the Participants According to Gender and the Disability Group (DOCX 20 kb)

Additional file 4: **Table S3.** Hazards Ratios for Improved Functional Ability Among Men Who Were Followed-Up for > 6 Months According to Socioeconomic Status (DOCX 16 kb)

Additional file 5: **Table S4.** Hazards Ratios for Improved Functional Ability Among Women Who Were Followed-Up for > 6 Months According to Socioeconomic Status (DOCX 16 kb)

## Abbreviations

JAGES: Japan Gerontological Evaluation Study
LTC: long-term care
LTCI: LTC insurance
SES: socioeconomic status
