# Supplementary material for: Socioeconomic status and improvement in functional ability among older adults in Japan: a longitudinal study
Source: BMC Public Health. 2019 Feb 19;19:209. doi: 10.1186/s12889-019-6531-9 (PMC6381753; doi:10.1186/s12889-019-6531-9)
Supplement: Supplementary file 1 — Survey Questionnaire (Japan Gerontological Evaluation Study, version 2010) (DOCX 43 kb) [file 12889_2019_6531_MOESM1_ESM.docx]

Additional File. Survey Questionnaire (Japan Gerontological Evaluation Study, version 2010)

| Q1. First we would like to ask you about your physical status. |
| --- |
| 1) How is your current health status? |
| 1. Excellent |
| 2. Good |
| 3. Fair |
| 4. Poor |
| 2) Can you walk, take a bath or use a toilet independently? |
| 1. Yes, without assistance |
| 2. Yes, with partial assistance. |
| 3. I need full assistance |
| 3) Are you currently receiving any medical treatment? |
| 1. Yes |
| 2. No |
| 4) If your answer was “1. Yes”, what is/are your illness(es) or disability(ies)? Circle the numbers of all the answers that apply. |
| 1. Cancer |
| 2. Heart disease (including arrhythmia) |
| 3. Stroke |
| 4. High blood pressure |
| 5. Diabetes (including mild type) |
| 6. Obesity |
| 7. Hyperlipidaemia |
| 8. Osteoporosis |
| 9. Joint disease / neuralgia |
| 10. Injury / fracture |
| 11. Respiratory disease |
| 12. Gastrointestinal disease |
| 13. Liver disease |
| 14. Mental disease |
| 15. Difficulty swallowing |
| 16. Impaired vision |
| 17. Impaired hearing |
| 18. Elimination problems (including incontinence, frequent urination, difficulty in starting urination, leaking of urine, etc.) |
| 19. Sleep problem |
| 20. Undiagnosed illness |
| 21.Other ( ) |
| 8) Have you ever had a check-up at a health center, your workplace, a medical institution, or another place? |
| 1. I had one within a year |
| 2. I had one within 2 to 3 years |
| 3. I had one more than 4 years ago |
| 4. I've never had one |
| Question 2. The next questions are about your dental health. |
| 1) What is the status of your dental health? |
| 1. I have 20 or more natural teeth |
| 2. I have 10 to 19 natural teeth |
| 3. I have 1 to 9 natural teeth |
| 4. I have no natural teeth |
| 2) Do you wear dentures or bridges (non-removable dentures) ? |
| 1. No |
| 2. Yes, in the upper jaw |
| 3. Yes, in the lower jaw |
| 4. Yes, in both jaws |
| Question 3. The next questions are about your dietary life. |
| 1) Have you lost more than 3 kg over the past six months? |
| 1. Yes |
| 2. No |
| 2) Do you find chewing hard food more difficult compared to half a year earlier? |
| 1. Yes |
| 2. No |
| 3) Have you ever choked on tea or soup? |
| 1. Yes |
| 2. No |
| 4) Are you bothered by a feeling of thirst? |
| 1. Yes |
| 2. No |
| 5) How many meals do you have a day? |
| 1. One |
| 2. Two |
| 3. Three |
| 4. Four or more |
| 6) Who do you usually have meals with? Circle the numbers of all the answers that apply. |
| 1. No one |
| 2. Spouse |
| 3. Children |
| 4. Grandchildren |
| 5. Friends |
| 6. Other |
| 7) Who do you usually drink alcohol with? Circle the numbers of all the answers that apply. |
| 1. No one |
| 2. Spouse |
| 3. Children |
| 4. Grandchildren |
| 5. Friends |
| 6. Other |
| 7. I don’t drink alcohol |
| 8)How often did you eat meat or fish over the past month? |
| 1. Twice a day or more |
| 2. Once a day |
| 3. Four to six times a week |
| 4. Two or three times a week |
| 5. Once a week |
| 6. Less than once a week |
| 7. None |
| 9)How often do you eat fruits and vegetables over the past month? |
| 1. Twice a day or more |
| 2. Once a day |
| 3. Four to six times a week |
| 4. Two or three times a week |
| 5. Once a week |
| 6. Less than once a week |
| 7. None |
| Question 4. The next questions are about alcohol drinking and smoking. |
| 1) Do you currently drink alcohol? |
| 1. Yes |
| 2. I used to drink |
| 3. No |
| 2) If your answer was “1. Yes” or “2. I used to drink”, how often do/did you drink alcohol? |
| 1. Almost everyday |
| 2. 5 to 6 days a week |
| 3. 3 to 4 days a week |
| 4. 1 to 2 days a week |
| 5. 1 to 3 days a month |
| 6. Less than 1 day a month |
| 3) If your answer is “2. I used to drink”, what were the reasons for you to stop drinking? |
| 1. I stopped drinking due to illness |
| 2. Other reasons except illness ( ) |
| 4) Do you smoke cigarettes? |
| 1. I have never smoked |
| 2. I stopped smoking 5 or more years ago |
| 3. I stopped smoking within the past 4 years |
| 4. I am currently a smoker |
| Q5. The following questions are about your daily life. Circle the number of the appropriate answer. |
| 1) Can you go out alone by train or bus? |
| 1. Yes |
| 2. No |
| 2) Can you go shopping for daily necessities? |
| 1. Yes |
| 2. No |
| 3) Can you cook for yourself? |
| 1. Yes |
| 2. No |
| 4) Can you pay your bills by yourself? |
| 1. Yes |
| 2. No |
| 5) Can you deposit or withdraw money from your bank / postal savings account(s) by yourself? |
| 1. Yes |
| 2. No |
| 6) Can you complete the paperwork for your pension, etc. by yourself? |
| 1. Yes |
| 2. No |
| 7) Do you read newspapers? |
| 1. Yes |
| 2. No |
| 8) Do you read books or magazines? |
| 1. Yes |
| 2. No |
| 9) Are you interested in health-related articles or TV programs? |
| 1. Yes |
| 2. No |
| 10) Do you visit your friends’ homes? |
| 1. Yes |
| 2. No |
| 11) Do you give advice to your family members or friends? |
| 1. Yes |
| 2. No |
| 12) Can you visit people who have fallen ill? |
| 1. Yes |
| 2. No |
| 13) Do you start conversations with young people? |
| 1. Yes |
| 2. No |
| 14) Do you walk without stopping for about 15 minutes? |
| 1. Yes |
| 2. No |
| 15) Do people around you notice your forgetfulness, for example, by telling you that you often ask the same thing? |
| 1. Yes |
| 2. No |
| 16) Do you look up phone numbers and make phone calls by yourself? |
| 1. Yes |
| 2. No |
| 17) Do you sometimes forget what date it is today? |
| 1. Yes |
| 2. No |
| Question 6. The following questions are about going out in daily life. |
| 1) How often do you go out? |
| 1. Almost everyday |
| 2. Two or three times a week |
| 3. Once a week |
| 4. Once or twice a month |
| 5. Several times a year |
| 6. Rarely |
| 2) Has the frequency of your going out decreased since last year? |
| 1. Yes |
| 2. No |
| 3) What do you use for transport when you go out? Circle the numbers of all the answers that apply. |
| 1. Driving by myself |
| 2. Family members’ car |
| 3. Friends’ car |
| 4. Train |
| 5. Bus |
| 6. Taxi |
| 7. Transport by volunteers |
| 8. Motorcycle |
| 9. Bicycle |
| 10. Other |
| 4) Have you had any falls over the past year? |
| 1. Many times |
| 2. Once |
| 3. None |
| 5) Are you very worried about falls? |
| 1. Yes |
| 2. No |
| 6) Do you go up stairs without holding onto the handrail or the wall? |
| 1. Yes |
| 2. No |
| 7) Do you get up out of chairs without holding anything? |
| 1. Yes |
| 2. No |
| 8) How long do you walk a day on average? |
| 1. Less than 30 minutes |
| 2. 30 to 59 minutes |
| 3. 60 to 89 minutes |
| 4. 90 minutes or more |
| Q7. The following questions are about your leisure activities. |
| 1) Do you engage in any leisure activities at the moment? |
| 1. Yes |
| 2. No |
| 2) If your answer was “1. Yes”, what are the leisure activities? Circle the numbers of all the answers that apply. |
| 1. Golf |
| 2. Ground golf |
| 3. Gateball [Japanese croquet] |
| 4. Calisthenics, tai chi chuan |
| 5. Walking, jogging |
| 6. Playing go [Japanese board game played with black and white stones] |
| 7. Reading |
| 8. Using the computer |
| 9. Playing musical instruments |
| 10. Choir, folk singing |
| 11. Karaoke |
| 12. Dancing |
| 13. Haiku [Japanese poetry consisting seventeen syllables], tanka [Japanese poetry consisting thirty-one syllables], senryu [Japanese poetry] |
| 14. Calligraphy |
| 15. Sado [Japanese tea ceremony], flower arrangement |
| 16. Crafts |
| 17. Painting |
| 18. Photography |
| 19. Horticulture, gardening |
| 20. Growing crops |
| 21. Travelling |
| 22. Mountain climbing |
| 23. Fishing |
| 24. Playing pachinko [Japanese pinball] |
| 25. Other ( ) |
| Question 8. How often do you attend activities for the following groups? Please circle the number next to the (most) appropriate answer in the following questions. |
| 1) Political organisation or group |
| 1. Almost everyday |
| 2. Two or three times a week |
| 3. Once a week |
| 4. Once or twice a month |
| 5. A few times a year |
| 6. Never |
| 2) Industrial or trade association |
| 1. Almost everyday |
| 2. Two or three times a week |
| 3. Once a week |
| 4. Once or twice a month |
| 5. A few times a year |
| 6. Never |
| 3) Volunteer group |
| 1. Almost everyday |
| 2. Two or three times a week |
| 3. Once a week |
| 4. Once or twice a month |
| 5. A few times a year |
| 6. Never |
| 4) Senior citizen club |
| 1. Almost everyday |
| 2. Two or three times a week |
| 3. Once a week |
| 4. Once or twice a month |
| 5. A few times a year |
| 6. Never |
| 5) Religious organisation or group |
| 1. Almost everyday |
| 2. Two or three times a week |
| 3. Once a week |
| 4. Once or twice a month |
| 5. A few times a year |
| 6. Never |
| 6) Sports group or club |
| 1. Almost everyday |
| 2. Two or three times a week |
| 3. Once a week |
| 4. Once or twice a month |
| 5. A few times a year |
| 6. Never |
| 7) Neighborhood association or residents’ association |
| 1. Almost everyday |
| 2. Two or three times a week |
| 3. Once a week |
| 4. Once or twice a month |
| 5. A few times a year |
| 6. Never |
| 8) Leisure activity group |
| 1. Almost everyday |
| 2. Two or three times a week |
| 3. Once a week |
| 4. Once or twice a month |
| 5. A few times a year |
| 6. Never |
| Question 9. The following questions are about your relationships with your friends. |
| 1) How often do you see your friends? |
| 1. Almost everyday |
| 2. Two or three times a week |
| 3. Once a week |
| 4. Once or twice a month |
| 5. A few times a year |
| 6. Rarely |
| 2) How many friends/acquaintances have you seen over the past month? Count the same person as one, no matter how many times you have seen him/her. |
| 1. None |
| 2. 1 to 2 |
| 3. 3 to 5 |
| 4. 6 to 9 |
| 5. 10 or more |
| 3) Who do you see often? Circle the numbers of all the answers that apply. |
| 1. Neighbor or other person living in the same area |
| 2. Childhood friend |
| 3. Friend from your school days |
| 4. Colleague or former colleague |
| 5. Friend with the same interest or leisure activity |
| 6. Friend in the same volunteer activity |
| 7. Other |
| 8. None |
| Question 10. The following questions are about mutual assistance with the people around you. Circle the numbers of all the answers that apply. |
| 1) Do you have someone who listens to your concerns and complaints? |
| 1. Spouse |
| 2. Children living together |
| 3. Children or relatives living apart |
| 4. Neighbor |
| 5. Friend |
| 6. Other |
| 7. None |
| 2) Do you listen to someone’s concerns and complaints? Circle the numbers of all the answers that apply. |
| 1. Spouse |
| 2. Children living together |
| 3. Children or relatives living apart |
| 4. Neighbor |
| 5. Friend |
| 6. Other |
| 7. None |
| 3) Do you have someone who looks after you when you are sick and confined to a bed for a few days? Circle the numbers of all the answers that apply. |
| 1. Spouse |
| 2. Children living together |
| 3. Children or relatives living apart |
| 5. Neighbor |
| 6. Friend |
| 6. Other |
| 7. None |
| 4) Do you look after someone when he/she is sick and confined to a bed for a few days? Circle the numbers of all the answers that apply. |
| 1. Spouse |
| 2. Children living together |
| 3. Children or relatives living apart |
| 4. Neighbor |
| 5. Friend |
| 6. Other |
| 7. None |
| Q11. The following questions are about the area where you live. |
| 1) Do you think people living in your area can be trusted in general? |
| 1. Very |
| 2. Moderately |
| 3. Neutral |
| 4. Slightly |
| 5. Not at all |
| 2) Do you think people living in your area try to help others in most situations? |
| 1. Very |
| 2. Moderately |
| 3. Neutral |
| 4. Slightly |
| 5. Not at all |
| 3) How attached are you to the area you live? |
| 1. Very |
| 2. Moderately |
| 3. Neutral |
| 4. Slightly |
| 5. Not at all |
| 4) Which of the following did you regard as the “area” when answering the above questions? |
| 1. Neighborhood |
| 2. Residents’ association/Neighbourhood association |
| 3. Elementary school district level |
| 4. Municipal level |
| 5. Prefectural level |
| 6. Other |
| 5) Which of the following have changes have you seen in the area you live? Circle the numbers of all the answers that apply. |
| 1. Revitalization of the local economy |
| 2. Depression of the local economy |
| 3. Deterioration of security |
| 4. Increase of newcomers |
| 5. Revival of local festivals |
| 6. Decline of the local festival |
| 7. Increase in unemployment |
| 8. Increase in poverty |
| 9. Deterioration of administrative services |
| 10. Improvement of administrative services |
| 11. Widening income disparity |
| 12. Declining interaction or activities among community residents |
| 13. Increasing interaction or activities among community residents |
| 14. No major changes |
| 6) Do you agree with making it a rule to offer half a day for the interests of the whole area but not for your own interests? |
| 1. I agree |
| 2. Neutral |
| 3. I disagree |
| 7) How uneasy do you feel about safety in your community? |
| 1. Very uneasy |
| 2. More or less uneasy |
| 3. Slightly uneasy |
| 4. Not uneasy at all |
| 8) Do you want to participate in local events or festivals positively? |
| 1. Yes |
| 2. No |
| 9) What kind of interactions do you have with people in your neighborhood? |
| 1. Mutual consultation, lending and borrowing daily commodities, cooperation in daily life |
| 2. Standing and chatting frequently |
| 3. No more than exchanging greetings |
| 4. None, not even greetings |
| Question 12. Are the following present within 1 km of your home? |
| 1) Locations with noticeable graffiti or undisposed garbage |
| 1. Many |
| 2. Some |
| 3. Few |
| 4. None |
| 5. I don’t know |
| 2) Parks or foot paths suitable for exercise or walking |
| 1. Many |
| 2. Some |
| 3. Few |
| 4. None |
| 5. I don’t know |
| 3) Locations difficult for walking, such as hills or steps |
| 1. Many |
| 2. Some |
| 3. Few |
| 4. None |
| 5. I don’t know |
| 4) Roads or crossroads with a great risk of traffic accidents |
| 1. Many |
| 2. Some |
| 3. Few |
| 4. None |
| 5. I don’t know |
| 5) Fascinating views or buildings |
| 1. Many |
| 2. Some |
| 3. Few |
| 4. None |
| 5. I don’t know |
| 6) Shops or facilities selling fresh fruits and vegetables |
| 1. Many |
| 2. Some |
| 3. Few |
| 4. None |
| 5. I don’t know |
| 7) Dangerous places when walking alone at night |
| 1. Many |
| 2. Some |
| 3. Few |
| 4. None |
| 5. I don’t know |
| 8) Houses or facilities you feel free to drop in |
| 1. Many |
| 2. Some |
| 3. Few |
| 4. None |
| 5. I don’t know |
| Q13. Circle the number of the appropriate answer in the following questions. |
| 1) Are you satisfied with your current life? |
| 1. Yes |
| 2. No |
| 2) Do you sometimes feel there is no point in living? |
| 1. Yes |
| 2. No |
| 3) Do you think your energy for daily life or your interest in what's going on in the world has been decreasing? |
| 1. Yes |
| 2. No |
| 4) Do you feel your life is empty? |
| 1. Yes |
| 2. No |
| 5) Do you often feel bored? |
| 1. Yes |
| 2. No |
| 6) Do you usually feel good? |
| 1. Yes |
| 2. No |
| 7) Do you feel something bad is going to happen? |
| 1. Yes |
| 2. No |
| 8) Do you think you are fortunate? |
| 1. Yes |
| 2. No |
| 9) Do you often feel helpless? |
| 1. Yes |
| 2. No |
| 10) Do you prefer staying at home to going out? |
| 1. Yes |
| 2. No |
| 11) Do you think you are more forgetful than others? |
| 1. Yes |
| 2. No |
| 12) Do you think life is wonderful? |
| 1. Yes |
| 2. No |
| 13) Do you feel full of energy? |
| 1. Yes |
| 2. No |
| 14) Do you think there is no hope in your life? |
| 1. Yes |
| 2. No |
| 15) Do you think others are better off than you are? |
| 1. Yes |
| 2. No |
| 16) Do you feel there was no self-fulfillment in everyday life? |
| 1. Yes |
| 2. No |
| 17) Has something you used to do easily become bothersome? |
| 1. Yes |
| 2. No |
| 18) Dose something you used to do easily become bothersome? |
| 1. Yes |
| 2. No |
| 19) Have you thought you are not useful? |
| 1. Yes |
| 2. No |
| 20) Have you felt tired for no reason? |
| 1. Yes |
| 2. No |
| Q14. Circle the number of the answer that best describes your feelings. |
| 1) Do you feel you are treated unfairly? |
| 1. Very often |
| 2. Occasionally |
| 3. Neutral |
| 4. Not often |
| 5. Never |
| 2) What you do everyday... |
| 1. Gives you pleasure and satisfaction |
| 2. Tends towards the above |
| 3. Neutral |
| 4. Tends towards the below |
| 5. Gives you no pleasure or satisfaction |
| 3) Does your feeling or thinking get very confused? |
| 1. Very often |
| 2. Occasionally |
| 3. Neutral |
| 4. Not often |
| 5. Never |
| 4) Do you experience undesired emotions? |
| 1. Very often |
| 2. Occasionally |
| 3. Neutral |
| 4. Not often |
| 5. Never |
| 5) Do you feel what you do everyday has little meaning to you? |
| 1. Very often |
| 2. Occasionally |
| 3. Neutral |
| 4. Not often |
| 5. Never |
| 6) Do you lose confidence in your ability to keep self-control? |
| 1. Very often |
| 2. Occasionally |
| 3. Neutral |
| 4. Not often |
| 5. Never |
| Q15. The following questions are about your personal characteristics. Circle the number of the answer the best applies. |
| 1) Sex |
| 1. Male |
| 2. Female |
| 2) Age |
| ( ) years old |
| 3) What is your current height and weight? (Give a rough estimate) |
| Height: Approximately ( ) cm |
| Weight: Approximately ( ) kg |
| 4) How long have you been living in ｘｘ City? |
| 1. Less than 5 years |
| 2. 5 to 9 years |
| 3. 10 to 19 years |
| 4. 20 to 29 years |
| 5. 30 to 39 years |
| 6. 40 to 49 years |
| 7. 50 years or more |
| 5) What prefecture did you live in when you were 15 years old? |
| ( ) |
| 6) How many years of formal education have you had? |
| 1. Less than 6 years |
| 2. 6 to 9 years |
| 3. 10 to 12 years |
| 4. 13 years or more |
| 5. Other |
| Question 16. The next questions are about your family. |
| 1) What is your marital status? |
| 1. Married |
| 2. Widowed |
| 3. Divorced |
| 4. Never married |
| 5. Other |
| 2) Who do you live with? Circle the numbers of all the answers that apply. |
| 1. I live alone |
| 2. Spouse |
| 3. Children |
| 4. Spouses of children |
| 5. Grandchildren or great-grandchildren |
| 6. Parents |
| 7. Parents-in-law |
| 8. Brothers or sisters |
| 9. Brothers/sisters-in-law |
| 10. Other relatives |
| 11. Unrelated person/people |
| 12. Other ( ) |
| 3) How many people live in your household? |
| ( ) people (including yourself) |
| 4) What was your pretax annual household income for 2009 (including pension)? Circle the number of the answer that best applies. |
| 1. Less than 500,000 yen |
| 2. 500,000 to less than 1 million yen |
| 3. 1 million to less than 1.5 million yen |
| 4. 1.5 million to less than 2 million yen |
| 5. 2 million to less than 2.5 million yen |
| 6. 2.5 million to less than 3 million yen |
| 7. 3 million to less than 4 million yen |
| 8. 4 million to less than 5 million yen |
| 9. 5 million to less than 6 million yen |
| 10. 6 million to less than 7 million yen |
| 11. 7 million to less than 8 million yen |
| 12. 8 million to less than 9 million yen |
| 13. 9 million to less than 10 million yen |
| 14. 10 million to less than 12 million yen |
| 15. More than 12 million yen |
| Question 17. The next questions are about your house. |
| 1) What type of residence do you live in? |
| 1. Owned house |
| 2. Privately-rented house |
| 3. Municipally-managed house |
| 4. Company-owned house |
| 5. Other |
| 2) What is the architectural type of your home? |
| 1. Detached house |
| 2. Row house |
| 3. Apartment building |
| 4. Other |
| Q18. Have you experienced any of the following events over the past year? Circle the numbers of all answers that apply. |
| 1. I started a new job. |
| 2. I quit my job or retired. |
| 3. I started living with my children. |
| 4. I started living alone. |
| 5. I became better off financially. |
| 6. I became worse off financially. |
| 7. A new grandchild or great-grandchild was born |
| 8. I lost my spouse. |
| 9. A family member or close friend or relative passed away |
| 10. I acquired new friends. |
| 11. I suffered a serious illnesses. |
| 12. I started caring for sick family members. |
| 13. Other ( ) |
| 14. No major changes. |
| Question 19. How worried are you if you have to deal with an unexpected expense? |
| 1. Not at all |
| 2. Slightly |
| 3. Moderately |
| 4. Very |
| Question 20. Are you receiving a pension? Please circle numbers next to all applicable answers. |
| 1. National pension |
| 2. Employee pension or mutual aid pension |
| 3. Pension fund; corporate pension |
| 4. Personal pension |
| 5. I am not receiving any pension. |
| Q21. The following questions are about your past and current jobs. |
| 1) What is your current working status? |
| 1. I have a paid job. |
| 2. I am retired from my job. |
| 3. I have never had a job |
| 2) What type of occupation have you been engaged in for the longest in your life? |
| 1. Specialist, technician |
| 2. Manager |
| 3. Clerical worker |
| 4. Sales/service jobs |
| 5. Labor |
| 6. Agriculture, forestry or fisheries |
| 7. Other ( ) |
| 8. I have never had a job |
| 3) How many people worked at the company or organization where you worked the longest? |
| 1. 1 to 9 people |
| 2. 10 to 49 people |
| 3. 50 to 499 people |
| 4. 500 to 9,999 people |
| 5. 10,000 people or more |
| 6. I don’t know |
| 7. I have never had a job |
| 4) (For those who currently have a job) How many hours are you working on average a week? |
| Approximately ( ) hours |
| Question 22. The next questions are about nursing care by the family. |
| 1) Do you provide nursing care to any of your family members? |
| 1. Yes |
| 2. No |
| 2) (For those caring for family members) Who are you caring for? |
| 1. Spouse |
| 2. Parent(s) |
| 3. Parent(s)-in-law |
| 4. Brothers or sisters |
| 5. Other ( ) |
| Question 23. The next questions are about medical treatment. |
| 1) How much do you rely on what physicians say? |
| 1. Very |
| 2. Moderately |
| 3. Neutral |
| 4. Slightly |
| 5. Not at all |
| 2) Do you have a regular doctor? |
| 1. Yes |
| 2. No |
| 3) Have you refused or discontinued medical treatment for your illness(es) or disability(ies) over the past year? |
| 1. Yes |
| 2. No |
| 4) If your answer was “1. Yes”, what is the reason? Circle the numbers of all the answers that apply. |
| 1. The waiting time is too long. |
| 2. The expenses are too high. |
| 3. There is no facility close to my home. |
| 4. I don’t know where to go. |
| 5. I have no means of transport. |
| 6. I don’t like seeing doctors. |
| 7. I am too busy to go. |
| 8. It is not serious enough to see a doctor. |
| 9. Other ( ) |
| (For those receiving medical treatment) |
| 5) Did your attending doctor listen to you talk about your symptoms? Please answer the question according to your last experience. |
| 1. Very |
| 2. Moderately |
| 3. Neutral |
| 4. Slightly |
| 5. Not at all |
| (For those receiving medical treatment) |
| 6) Was the explanation from the attending doctor easy to understand? Please answer the question according to your last experience. |
| 1. Very |
| 2. Moderately |
| 3. Neutral |
| 4. Slightly |
| 5. Not at all |
| Question 24. The next questions are about your life style. |
| 1) How many percent have you turned out for national or local government elections over the past three years? |
| 1. All |
| 2. More than half |
| 3. Less than half |
| 4. I am interested in them but have never voted. |
| 5. I am not interested in them so have never voted. |
| 2) Which of the following do you need (desire to have) but you don’t have due to financial or familial reasons? Please circle numbers next to all applicable answers. |
| 1. TV set |
| 2. Refrigerator |
| 3. Air conditioner; heater; kotatsu [Japanese table with a heat source] |
| 4. Microwave oven |
| 5. Water heater |
| 6. Telephone (including that with fax function) |
| 7. Ceremonial dress |
| 3) Please circle numbers next to all items which you have in your current house. ※ “Private” means you don’t share it with other households. |
| 1. Private toilet |
| 2. Private kitchen |
| 3. Private bathroom |
| 4. Private washroom separated from kitchen |
| 5. Dining room separated from bedroom |
| 4) Haven’t you been able to attend any ceremonial occasions of your relatives’ due to the tips or transport expenses over the past years? |
| 1 Yes |
| 2. No |
| 3. No ceremonial occasions to attend |
| 5) Have you ever experienced cut-off of water, electricity, gas, telephone, cell phone or other services, because you didn’t pay the bill? (except that you forgot to pay) |
| 1. Yes |
| 2. No |
| **Question 22. The following questions are about your dental and oral care.** |
| 1) Have you visited the dentist in the past six months for treatment (including denture adjustment)? |
| 1. Yes |
| 2. No |
| 2) Have you visited the dentist in the past six months for anything other than treatment (e.g. routine dental checkup)? |
| 1. Yes |
| 2. No |
| 3) Do you use inter-dental brushes and/or dental floss? |
| 1. No |
| 2. Sometimes |
| 3. Every day |
| 4) Do you use toothpaste with fluoride? |
| 1. Yes |
| 2. I use toothpaste, but I don't know if it has fluoride |
| 3. No |
| 5) How healthy is your oral cavity (i.e. teeth, gums, dentures)? |
| 1. Very healthy |
| 2. Healthy |
| 3. Not very healthy |
| 4. Not healthy |
| 6) Have you experienced any problems with your teeth, gums, and/or dentures in the past six months? Circle all that apply. |
| 1. Difficulty eating |
| 2. Difficulty speaking |
| 3. Hesitated showing teeth when laughing or talking |
| 4. Felt more irritable (uneasy) than usual |
| 5. Could not enjoy myself when around family, friends, neighbors, or other people |
| 6. No problems |
| 7) How well can you eat hard food? |
| 1. I can chew and eat anything I want. |
| 2. I have trouble chewing some foods, but I can eat most foods. |
| 3. I can't chew well, and can only eat limited foods. |
| 4. I can hardly chew at all. |
| 5. I cannot chew at all and am on a liquid diet. |
| Question 23. Circle the number of the one answer that best applies. |
| 1) When future events are unclear, I predict the best. |
| 1. Strongly agree |
| 2. Agree |
| 3. Neutral |
| 4. Disagree |
| 5. Strongly disagree |
| 2) When it seems like something bad will happen, it usually does. |
| 1. Strongly agree |
| 2. Agree |
| 3. Neutral |
| 4. Disagree |
| 5. Strongly disagree |
| 3) I am always optimistic about my future. |
| 1. Strongly agree |
| 2. Agree |
| 3. Neutral |
| 4. Disagree |
| 5. Strongly disagree |
| 4) I rarely think that things will go my way. |
| 1. Strongly agree |
| 2. Agree |
| 3. Neutral |
| 4. Disagree |
| 5. Strongly disagree |
| 5) I rarely expect anything good to happen to me. |
| 1. Strongly agree |
| 2. Agree |
| 3. Neutral |
| 4. Disagree |
| 5. Strongly disagree |
| 6) I think that overall, more good things happen to me than bad things. |
| 1. Strongly agree |
| 2. Agree |
| 3. Neutral |
| 4. Disagree |
| 5. Strongly disagree |
| **Question 24. To what degree do you feel that you are happy?** |
| How happy are you, with 10 points for extremely happy and 1 point for very unhappy? Circle the number of the appropriate score. |
| 10 points Extremely happy 9 8 7 6 5 4 3 2 1 point Very unhappy |
| Question 22. Do you conduct activities or play a role in the area you live in? Circle all that apply. |
| 1. Residents' association official |
| 2. Welfare commissioners |
| 3. Child welfare comissioners |
| 4. Women's association |
| 5. Help people in the neighborhood |
| 6. NPO volunteer activities |
| 7. NPO volunteer executive member |
| 8. Interest or sports group official |
| 9. Festivals and other community events |
| 10. Other |
| 11. None in particular |
| 2) Consider a hypothetical situation where you lost your wallet with your address in it. If someone living in this area picked it up in the street, how likely would they be to return it to you untouched? |
| 1. Extremely likely |
| 2. Very likely |
| 3. Not very likely |
| 4. Very unlikely |
| 3) Do you feel that people can generally be trusted? |
| 1. Yes |
| 2. No |
| 3. It depends |
| 4) People in my area lock their doors when they go out. |
| 1. Strongly agree |
| 2. Agree |
| 3. Neutral |
| 4. Disagree |
| 5) The word "outsider," referring to people not from around here, is still in use here. |
| 1. Strongly agree |
| 2. Agree |
| 3. Neutral |
| 4. Disagree |
| 6) If a fight broke out among people in this area, it could be resolved by the people in this area. |
| 1. Strongly agree |
| 2. Agree |
| 3. Neutral |
| 4. Disagree |
| 5. Strongly disagree |
| 7) The area I live in is more cohesive than other areas. |
| 1. Strongly agree |
| 2. Agree |
| 3. Neutral |
| 4. Disagree |
| 5. Strongly disagree |
| 8) How is your relationship with your neighbors in your area? |
| 1. I am personally acquainted with and/or interact with almost everyone in my area. |
| 2. I am personally acquainted with and/or interact with about half the people in my area. |
| 3. I am personally acquainted with and/or interact with very few people in my area. |
| 4. No interaction |
| 9) In the past three years, how often did you give donations to or raise funds for an organization (e.g. social welfare council or NPO) working for the benefit of the area in which you live? |
| 1. Ten times or more |
| 2. A few times |
| 3. Once |
| 4. Never |
| Question 23. In the past year, did you experience any of the following acts by someone? |
| 1) Physical violence, such as being hit, kicked, having objects thrown at you, or being shut in a room. |
| 1. Never |
| 2. Once or twice |
| 3. Occasionally |
| 4. Frequently |
| 2) Acts to harm your self-esteem, such as verbal abuse, cutting remarks, or being ignored for long periods. |
| 1. Never |
| 2. Once or twice |
| 3. Occasionally |
| 4. Frequently |
| 3) If you answered yes ("once or twice," "occasionally," or "frequently") to question 1) or 2) above, did you consult anyone regarding the incident? Circle all that apply. |
| 1. Friend or acquaintance |
| 2. Immediate or extended family member |
| 3. Doctor |
| 4. Police officer |
| 5. Other |
| 6. No consultation |
| 4) Does anyone (including a member of your family) take or use your savings or pension benefits without your consent? |
| 1. Yes |
| 2. No |
| 5) If you answered "Yes" to question 4), who used or took your savings or pension benefits? Circle all that apply. |
| 1. Family member that lives with me |
| 2. Immediate or extended family member that does not live with me |
| 3. Neighbor or acquaintance |
| 4. Other |
| 5. I don't know |
| **Question 22. What is your standard of living as seen from the general public?** |
| 1. Upper class |
| 2. Upper middle class |
| 3. Middle class |
| 4. Lower middle class |
| 5. Lower class |
| **Question 23. The following questions are about your regular sleeping habits over the past month.** |
| 1) How many actual hours of sleep did you get in the past month? This may be different from the number of hours you were in bed. |
| ___ hours and |
| ___ min a day on average |
| 2) How often did you experience the events below in the past month? Circle the answer that best applies. |
| ① You could not fall asleep within half an hour of going to bed. |
| 1. Never |
| 2. Less than once a week |
| 3. 1-2 times a week |
| 4. 3 or more times a week |
| ② You woke up in the middle of the night or early in the morning. |
| 1. Never |
| 2. Less than once a week |
| 3. 1-2 times a week |
| 4. 3 or more times a week |
| ③ You didn't feel well-rested when you woke up in the morning. |
| 1. Never |
| 2. Less than once a week |
| 3. 1-2 times a week |
| 4. 3 or more times a week |
| 3) How would you rate your sleep quality as a whole over the past month? |
| 1. Very good |
| 2. Good |
| 3. Poor |
| 4. Very poor |
| 4) How often in the past month did you take medication to help you sleep (that was prescribed by a doctor or that you bought at a pharmacy or other vendor)? |
| 1. Never |
| 2. Less than once a week |
| 3. 1-2 times a week |
| 4. 3 or more times a week |
| **Question 24. Do you currently experience difficulties because of forgetfulness in your daily life? Circle all below that apply.** |
| 1) Forget someone's name. |
| 1. Never |
| 2. Very rarely |
| 3. Occasionally |
| 4. Frequently |
| 2) Forget where you put something (or put something away). |
| 1. Never |
| 2. Very rarely |
| 3. Occasionally |
| 4. Frequently |
| 3) Forget to do something you were going to (planning to) do. |
| 1. Never |
| 2. Very rarely |
| 3. Occasionally |
| 4. Frequently |
| 4) Be unsure of the current date. |
| 1. Never |
| 2. Very rarely |
| 3. Occasionally |
| 4. Frequently |
| 5) Do you experience difficulties because of that forgetfulness? Circle all that apply. |
| 1. Quickly forget things you have just done or said or things that just happened. |
| 2. Are careless about the stove, faucet, or light switches. |
| 3. Forget to lock the door. |
| 4. Other |
| 5. Never |
| **Question 25. The following questions are about your past.** |
| 1) How many siblings do you have, and what number child are you? |
| Number _____ among |
| ______ children |
| 2) Did your family own their own house when you were 15? |
| 1. Yes |
| 2. No |
| 3) What was your standard of living when you were 15 as seen from the general public? |
| 1. Upper class |
| 2. Upper middle class |
| 3. Middle class |
| 4. Lower middle class |
| 5. Lower class |
| Question 26. How often do you take a bath in the bathtub? |
| Summer: ___ times a week |
| Winter: ___ times a week |
| Version E |
| 5) Which of the following numbers best applies to your work conditions? |
| 1. I spend a lot of time sitting in my job. |
| 2. I spend a lot of time standing or walking in my job, but the work does not require physical effort (e.g. shop assistant, hairdresser, security guard). |
| 3. My job requires some physical effort, such as carrying things or using tools (e.g. plumber, nurse, janitor). |
| 4. My job requires intense physical exertion, such as carrying heavy objects (e.g. construction worker). |
| **Question 25. The following questions are about your physical movement in regular daily life (exercise, sports, activities or housework requiring physical effort). *Do not include movement for work.** |
| 1) Do you exert yourself strenuously, to the extent of the following activities? |
| 1. Almost never |
| 2. One to three times a month |
| 3. About once a week |
| 4. Twice a week or more |
| 2) Do you exert yourself moderately, to the extent of the following activities? |
| 1. Almost never |
| 2. One to three times a month |
| 3. About once a week |
| 4. Twice a week or more |
| 3) Do you exert yourself lightly, to the extent of the following activities? |
| 1. Almost never |
| 2. One to three times a month |
| 3. About once a week |
| 4. Twice a week or more |
| 4) Do you do strength training or other exercise to maintain or increase your muscle strength (muscle mass)? *Includes sit-ups, squats, and leg bends and stretches. |
| 1. Almost never |
| 2. One to three times a month |
| 3. About once a week |
| 4. Twice a week or more |
| 5) Do you stretch (muscle stretching, calisthenics) to stretch or relieve tension in your body? |
| 1. Almost never |
| 2. One to three times a month |
| 2. One to three times a month |
| 4. Twice a week or more |
| 6) What types of exercise do you do? Circle all that apply. |
| 1. Walking |
| 2. Physical exercises |
| 3. Ground golf |
| 4. Strength training |
| 5. Cycling |
| 6. Golf |
| 7. Mountain climbing, hiking |
| 8. Bowling |
| 9. Swimming |
| 10. Jogging, running |
| 11. Stretching (calisthenics) |
| 12. Other ( ) |
| 7) Which of the following numbers best applies to you right now? "Regular exercise" means exercise for 20 minutes or longer at least once a week. |
| 1. I do not do any particular exercise currently, and do not plan to in the future. |
| 2. I do not do any particular exercise currently, but plan to in the future (in the next six months). |
| 3. I do a little exercise, but it is not regular. |
| 4. I recently (in the past six months) started exercising regularly, and still do now. |
| 5. I have been exercising regularly for more than six months. |
| 8) Do you ever exercise by yourself? |
| 1. Almost never |
| 2. One to three times a month |
| 3. About once a week |
| 4. Twice a week or more |
| 9) Do you ever exercise with family, friends, or acquaintances? |
| 1. Almost never |
| 2. One to three times a month |
| 3. About once a week |
| 4. Twice a week or more |
| 10) Do you ever take part in leisure activities (excluding exercise) with family, friends, or acquaintances? |
| 1. Almost never |
| 2. One to three times a month |
| 3. About once a week |
| 4. Twice a week or more |
| 11) How much TV (including videos) do you watch in a day on average? |
| 1. Less than one hour |
| 2. 1-2 hours |
| 3. 2-3 hours |
| 4. 3-4 hours |
| 5. 4 or more hours |
