# Supplementary material for: Socioeconomic status and improvement in functional ability among older adults in Japan: a longitudinal study
Source: BMC Public Health. 2019 Feb 19;19:209. doi: 10.1186/s12889-019-6531-9 (PMC6381753; doi:10.1186/s12889-019-6531-9)
Supplement: Supplementary file 2 — Table S1. Japan’s Long-Term Care System: Criteria and Benefit Limits (Standard Amounts) for In-Home Services (DOCX 15 kb) [file 12889_2019_6531_MOESM2_ESM.docx]

**Table S1. Japan’s Long-Term Care System: Criteria and Benefit Limits (Standard Amounts) for In-Home Services**

| **Disability Level** | **ADL Criteria** | **Available Services** | **Benefit Limit per Month (JP¥)^a,b^** |
| --- | --- | --- | --- |
| Requiring support-1/2 | LTC is needed for some aspects of daily living, but proper care can improve or maintain ADLs | LTC prevention programs | 49,700/104,000 |
| Requiring LTC-1 | Unstable in rising and gait. Partial support needed for toileting, bathing, dressing, and all other basic ADLs | Home-visit care. Facility-based services | 165,800 |
| Requiring LTC-2 | Difficulty in rising and gait. Partial or complete support needed for toileting, bathing, dressing, and all other basic ADLs | Home-visit care ≤3 times per week or facility-based services | 194,800 |
| Requiring LTC-3 | Inability to rise and no gait. Complete support needed in toileting, bathing, dressing, and all other basic ADLs | Home-visit care during the day and evening, home-visit intensive nursing care, or facility rehabilitation services (1–2 service times per day) | 267,500 |
| Requiring LTC-4 | Severe decline in ADL capacity. Complete support needed for toileting, bathing, dressing, and all other basic ADLs | Home-visit care during the day and evening, home-visit intensive nursing care, or facility rehabilitation services (2–3 service times per day) | 306,000 |
| Requiring LTC-5 | Complete support needed for all ADLs. Difficulty in communicating | Home-visit care during the day and evening, home-visit intensive nursing care, or facility rehabilitation services (3–4 service times per day) | 358,300 |

^a^Benefit limit = Standard amounts for in-home services per month. Japan’s LTC Insurance System pays 90.0% of the costs. The remaining 10.0% is paid by the user. Amounts vary slightly according to region.

^b^1,000 JP¥ = 9.0 US dollars (2017)
