# Supplementary material for: Socioeconomic status and improvement in functional ability among older adults in Japan: a longitudinal study
Source: BMC Public Health. 2019 Feb 19;19:209. doi: 10.1186/s12889-019-6531-9 (PMC6381753; doi:10.1186/s12889-019-6531-9)
Supplement: Supplementary file 3 — Table S2. Characteristics of the Participants According to Gender and the Disability Group (DOCX 20 kb) [file 12889_2019_6531_MOESM3_ESM.docx]

**Table S2. Clinical Characteristics of the Participants According to Gender and the Disability Group**

| **Characteristic** | **Disability Group at the Time of the Initial Assessment** | | | | | |
| --- | --- | --- | --- | --- | --- | --- |
|  | **Mild** | | **Moderate** | | **Severe** | |
|  | **All** | **Improved Functional Ability** | **All** | **Improved**  **Functional Ability** | **All** | **Improved Functional Ability** |
| **Men** | **n = 664** | **n = 74 (11.1%)** | **n = 789** | **n = 169 (21.4%)** | **n = 484** | **n = 103 (21.3%)** |
| Education (years), n (%) |  |  |  |  |  |  |
| ≤9 | 338 | 37 (10.9) | 393 | 86 (21.9) | 242 | 45 (18.6) |
| 10–12 | 154 | 13 (8.4) | 185 | 41 (22.2) | 108 | 19 (17.6) |
| 13+ | 92 | 13 (14.1) | 123 | 26 (21.1) | 75 | 25 (33.3) |
| Unknown | 80 | 11 (13.8) | 88 | 16 (18.2) | 59 | 14 (23.7) |
| Income (quartiles), n (%) |  |  |  |  |  |  |
| Q1 (lowest) | 112 | 12 (10.7) | 102 | 25 (24.5) | 82 | 15 (18.3) |
| Q2 | 158 | 15 (9.5) | 170 | 36 (21.2) | 105 | 24 (22.9) |
| Q3 | 118 | 10 (8.5) | 148 | 29 (19.6) | 81 | 15 (18.5) |
| Q4 (highest) | 115 | 16 (13.9) | 138 | 31 (22.5) | 94 | 21 (22.3) |
| Unknown | 161 | 21 (13.0) | 231 | 48 (20.8) | 122 | 28 (23.0) |
| Occupation, n (%) |  |  |  |  |  |  |
| Manual | 246 | 32 (13.0) | 272 | 69 (25.4) | 176 | 34 (19.3) |
| Non-manual | 217 | 26 (12.0) | 288 | 58 (20.1) | 161 | 40 (24.8) |
| Unknown | 201 | 16 (8.0) | 229 | 42 (18.3) | 147 | 29 (19.7) |
| Marital status, n (%)^a^ |  |  |  |  |  |  |
| Single/divorced/widowed | 112 | 16 (14.3) | 118 | 29 (24.6) | 66 | 19 (28.8) |
| Married/cohabiting | 498 | 49 (9.8) | 608 | 129 (21.2) | 377 | 76 (20.2) |
| Unknown | 54 | 9 (16.7) | 63 | 11 (17.5) | 41 | 8 (19.5) |
| Living alone, n (%) |  |  |  |  |  |  |
| N | 539 | 53 (9.8) | 666 | 146 (21.9) | 413 | 84 (20.3) |
| Y | 63 | 10 (15.9) | 48 | 8 (16.7) | 25 | 9 (36.0) |
| Unknown | 62 | 11 (17.7) | 75 | 15 (20.0) | 46 | 10 (21.7) |
| Comorbidity, n (%)^b^ |  |  |  |  |  |  |
| 0 | 159 | 13 (8.2) | 200 | 44 (22.0) | 108 | 27 (25.0) |
| ≥1 | 384 | 46 (12.0) | 465 | 99 (21.3) | 300 | 63 (21.0) |
| Unknown | 121 | 15 (12.4) | 124 | 26 (21.0) | 76 | 13 (17.1) |
| Depressive symptoms, n (%) |  |  |  |  |  |  |
| N | 276 | 28 (10.1) | 305 | 71 (23.3) | 208 | 40 (19.2) |
| Y | 234 | 20 (8.5) | 294 | 63 (21.4) | 153 | 33 (21.6) |
| Unknown | 154 | 26 (16.9) | 190 | 35 (18.4) | 123 | 30 (24.4) |
| **Women** | **n = 989** | **n = 120 (12.1%)** | **n = 816** | **n = 222 (27.2%)** | **n = 407** | **n = 123 (30.2%)** |
| Education (years), n (%) |  |  |  |  |  |  |
| ≤9 | 539 | 70 (13.0) | 472 | 132 (28.0) | 243 | 71 (29.2) |
| 10–12 | 241 | 29 (12.0) | 183 | 45 (24.6) | 74 | 25 (33.8) |
| 13+ | 82 | 7 (8.5) | 59 | 18 (30.5) | 29 | 11 (37.9) |
| Unknown | 127 | 14 (11.0) | 102 | 27 (26.5) | 61 | 16 (26.2) |
| Income (quartiles), n (%) |  |  |  |  |  |  |
| Q1 (lowest) | 206 | 22 (10.7) | 156 | 47 (30.1) | 81 | 21 (25.9) |
| Q2 | 148 | 20 (13.5) | 117 | 29 (24.8) | 42 | 15 (35.7) |
| Q3 | 128 | 13 (10.2) | 115 | 30 (26.1) | 50 | 12 (24.0) |
| Q4 (highest) | 145 | 20 (13.8) | 130 | 32 (24.6) | 79 | 31 (39.2) |
| Unknown | 362 | 45 (12.4) | 298 | 84 (28.2) | 155 | 44 (28.4) |
| Occupation, n (%) |  |  |  |  |  |  |
| Manual | 394 | 45 (11.4) | 314 | 87 (27.7) | 149 | 49 (32.9) |
| Non-manual | 214 | 23 (10.7) | 177 | 45 (25.4) | 88 | 27 (30.7) |
| Unknown | 381 | 52 (13.6) | 325 | 90 (27.7) | 170 | 47 (27.6) |
| Marital status, n (%)^a^ |  |  |  |  |  |  |
| Single/divorced/widowed | 496 | 63 (12.7) | 437 | 127 (29.1) | 225 | 65 (28.9) |
| Married/cohabiting | 403 | 48 (11.9) | 307 | 76 (24.8) | 137 | 46 (33.6) |
| Unknown | 90 | 9 (10.0) | 72 | 19 (26.4) | 45 | 12 (26.7) |
| Living alone, n (%) |  |  |  |  |  |  |
| N | 728 | 92 (12.6) | 615 | 160 (26.0) | 320 | 100 (31.3) |
| Y | 176 | 21 (11.9) | 134 | 43 (32.1) | 44 | 12 (27.3) |
| Unknown | 85 | 7 (8.2) | 67 | 19 (28.4) | 43 | 11 (25.6) |
| Comorbidity, n (%)^b^ |  |  |  |  |  |  |
| 0 | 253 | 33 (13.0) | 213 | 67 (31.5) | 96 | 35 (36.5) |
| ≥1 | 535 | 74 (13.8) | 485 | 129 (26.6) | 239 | 63 (26.4) |
| Unknown | 201 | 13 (6.5) | 118 | 26 (22.0) | 72 | 25 (34.7) |
| Depressive symptoms, n (%) |  |  |  |  |  |  |
| N | 388 | 43 (11.1) | 283 | 83 (29.3) | 145 | 49 (33.8) |
| Y | 314 | 34 (10.8) | 305 | 77 (25.2) | 127 | 36 (28.3) |
| Unknown | 287 | 43 (15.0) | 228 | 62 (27.2) | 135 | 38 (28.1) |

^a^Single = never married

^b^Comorbidity = heart disease, stroke, diabetes, or hypertension

N, no; Q, quartile; Y, yes
