# Supplementary material for: Socioeconomic status and improvement in functional ability among older adults in Japan: a longitudinal study
Source: BMC Public Health. 2019 Feb 19;19:209. doi: 10.1186/s12889-019-6531-9 (PMC6381753; doi:10.1186/s12889-019-6531-9)
Supplement: Supplementary file 4 — Table S3. Hazards Ratios for Improved Functional Ability Among Men Who Were Followed-Up for > 6 Months According to Socioeconomic Status (DOCX 16 kb) [file 12889_2019_6531_MOESM4_ESM.docx]

**Table S3. Hazards Ratios for Improved Functional Ability Among Men Who Were Followed-Up for >6 Months According to Socioeconomic Status**

| **Socioeconomic Factor** | **Disability Group at the Time of the Initial Assessment** | | | | | |
| --- | --- | --- | --- | --- | --- | --- |
|  | **Mild (n = 419)** | | **Moderate (n = 397)** | | **Severe (n = 167)** | |
|  | **Crude** | **Model^a^** | **Crude** | **Model^a^** | **Crude** | **Model^a^** |
|  | **HR (95.0% CI)** | **HR (95.0% CI)** | **HR (95.0% CI)** | **HR (95.0% CI)** | **HR (95.0% CI)** | **HR (95.0% CI)** |
| Education (years) |  |  |  |  |  |  |
| ≤9 | Ref. | Ref. | Ref. | Ref. | Ref. | Ref. |
| 10–12 | 0.47 (0.10–2.19) | 0.19 (0.03–1.46) | 0.76 (0.36–1.62) | 0.60 (0.26–1.38) | 2.25 (0.89–5.68)^+^ | 6.09 (1.83–20.33)^**^ |
| 13+ | 0.95 (0.21–4.42) | 1.77 (0.28–11.28) | 0.72 (0.31–1.67) | 0.58 (0.22–1.50) | 2.40 (0.80–7.16) | 2.43 (0.50–11.92) |
| Unknown | 2.30 (0.82–6.47) | 1.52 (0.18–13.25) | 0.68 (0.24–1.94) | 0.67 (0.13–3.40) | 0.86 (0.19–3.98) | 2.43 (0.16–36.57) |
| Income (quartiles) |  |  |  |  |  |  |
| Q1 (lowest) | Ref. | Ref. | Ref. | Ref. | Ref. | Ref. |
| Q2 | 0.48 (0.08–2.88) | 0.50 (0.07–3.70) | 0.53 (0.21–1.34) | 0.36 (0.12–1.02)^+^ | 2.57 (0.71–9.21) | 1.88 (0.37–9.67) |
| Q3 | 0.00 (0.00–0.00) | 0.00 (0.00–0.00) | 0.77 (0.32–1.85) | 0.89 (0.32–2.47) | 1.12 (0.25–5.04) | 0.49 (0.07–3.61) |
| Q4 (highest) | 1.51 (0.34–6.73) | 3.34 (0.55–20.35) | 0.40 (0.14–1.14)^+^ | 0.25 (0.08–0.79)^*^ | 0.64 (0.11–3.85) | 0.28 (0.03–2.36) |
| Unknown | 2.24 (0.62–8.14) | 3.31 (0.59–18.67) | 0.52 (0.22–1.26) | 0.40 (0.15–1.08) | 0.92 (0.22–3.86) | 1.51 (0.26–8.82) |
| Occupation |  |  |  |  |  |  |
| Manual | Ref. | Ref. | Ref. | Ref. | Ref. | Ref. |
| Non-Manual | 1.49 (0.50–4.42) | 5.09 (1.27–20.45)^*^ | 0.55 (0.28–1.11)^+^ | 0.55 (0.25–1.24) | 1.78 (0.73–4.35) | 1.16 (0.38–3.61) |
| Unknown | 1.38 (0.45–4.29) | 2.82 (0.47–16.98) | 0.79 (0.39–1.60) | 0.47 (0.16–1.41) | 0.88 (0.29–2.68) | 0.50 (0.08–3.31) |

^**^*P* < .01, ^*^*P* < .05, ^+^*P* < .10

^a^Adjusted for age, other socioeconomic status, marital status, living status, comorbidities, depressive symptoms, and municipality

CI, confidence interval; HR, hazard ratio; Q, quartile; Ref., reference
