# Supplementary material for: Socioeconomic status and improvement in functional ability among older adults in Japan: a longitudinal study
Source: BMC Public Health. 2019 Feb 19;19:209. doi: 10.1186/s12889-019-6531-9 (PMC6381753; doi:10.1186/s12889-019-6531-9)
Supplement: Supplementary file 5 — Table S4. Hazards Ratios for Improved Functional Ability Among Women Who Were Followed-Up for > 6 Months According to Socioeconomic Status (DOCX 16 kb) [file 12889_2019_6531_MOESM5_ESM.docx]

**Table S4. Hazards Ratios for Improved Functional Ability Among Women Who Were Followed-Up for >6 Months According to Socioeconomic Status**

| **Socioeconomic Factor** | **Disability Group at the Time of the Initial Assessment** | | | | | |
| --- | --- | --- | --- | --- | --- | --- |
|  | **Mild (n = 672)** | | **Moderate (n = 429)** | | **Severe (n = 171)** | |
|  | **Crude** | **Model^a^** | **Crude** | **Model^a^** | **Crude** | **Model^a^** |
|  | **HR (95.0% CI)** | **HR (95.0% CI)** | **HR (95.0% CI)** | **HR (95.0% CI)** | **HR (95.0% CI)** | **HR (95.0% CI)** |
| Education (years) |  |  |  |  |  |  |
| ≤9 | Ref. | Ref. | Ref. | Ref. | Ref. | Ref. |
| 10–12 | 0.88 (0.37–2.10) | 0.87 (0.33–2.29) | 1.50 (0.77–2.93) | 1.63 (0.75–3.56) | 2.05 (0.82–5.16) | 4.66 (1.36–16.00)^*^ |
| 13+ | 0.39 (0.05–2.92) | 0.49 (0.06–3.99) | 2.64 (1.14–6.08)^*^ | 4.22 (1.47–12.13) | 5.07 (1.77–14.53)^**^ | 7.12 (1.16–43.83)^*^ |
| Unknown | 0.80 (0.24–2.71) | 0.92 (0.19–4.60) | 0.75 (0.29–1.94) | 0.68 (0.13–3.59) | 0.75 (0.21–2.65) | 3.09 (0.42–22.61) |
| Income (quartiles) |  |  |  |  |  |  |
| Q1 (lowest) | Ref. | Ref. | Ref. | Ref. | Ref. | Ref. |
| Q2 | 0.19 (0.02–1.57) | 0.18 (0.02–1.59) | 0.60 (0.26–1.34) | 0.52 (0.21–1.33) | 1.87 (0.45–7.84) | 2.32 (0.33–16.13) |
| Q3 | 1.03 (0.29–3.67) | 1.18 (0.30–4.62) | 0.36 (0.12–1.11) | 0.25 (0.07–0.85)^*^ | 0.50 (0.06–4.31) | 0.28 (0.02–4.22) |
| Q4 (highest) | 1.07 (0.33–3.49) | 1.07 (0.30–3.89) | 0.30 (0.10–0.92)^*^ | 0.23 (0.07–0.80)^*^ | 2.16 (0.71–6.62) | 1.74 (0.36–8.47) |
| Unknown | 1.14 (0.43–2.99) | 1.04 (0.36–3.03) | 0.54 (0.27–1.07)^+^ | 0.53 (0.22–1.26) | 1.51 (0.53–4.35) | 1.48 (0.33–6.59) |
| Occupation |  |  |  |  |  |  |
| Manual | Ref. | Ref. | Ref. | Ref. | Ref. | Ref. |
| Non-Manual | 1.22 (0.47–3.14) | 1.42 (0.51–3.95) | 0.97 (0.43–2.17) | 0.90 (0.34–2.33) | 0.81 (0.34–1.93) | 0.19 (0.04–0.81)^*^ |
| Unknown | 1.01 (0.44–2.33) | 0.85 (0.32–2.25) | 1.46 (0.79–2.70) | 0.86 (0.38–1.97) | 0.56 (0.22–1.42) | 0.32 (0.07–1.42) |

^**^*P* < .01, ^*^*P* < .05, ^+^*P* < .10

^a^Adjusted for age, other socioeconomic status, marital status, living status, comorbidities, depressive symptoms, and municipality

CI, confidence interval; HR, hazard ratio; Q, quartile; Ref., reference
